# COVID-19 in the Perioperative Period of Cardiovascular Surgery: An Institutional Report

**DOI:** 10.21470/1678-9741-2022-0253

**Published:** 2023-06-14

**Authors:** Luisa Schilmann Frisso, Ana Carolina Simões Ramos, Assad Miguel Sassine

**Affiliations:** 1 Department of Cardiovascular Surgery, Escola Superior de Ciências da Santa Casa de Misericórdia de Vitória, Vitória, Espírito Santo, Brazil; 2 Teaching, Research and Innovation Center, Fundação Estadual de Inovação em Saúde (iNOVA Capixaba), Vila Velha, Espírito Santo, Brazil; 3 Department of Cardiovascular Surgery, Hospital Santa Casa de Misericórdia de Vitória, Vitória, Espírito Santo, Brazil. E-mail: assadsassine@gmail.com

Dear editor,

In our institution, during the recent coronavirus disease 2019 (COVID-19) pandemic, we had to prioritize assistance for patients with severe COVID-19. We tried to gather resources and staff to fuel the intensive care unit (ICU). Some surgeries were canceled, others were rescheduled, and some patients stayed at home awaiting their time to get their treatment. Aside, some cardiovascular services worked with urgency and emergence protocols, but no experience of COVID-19 and cardiovascular surgery had previously been reported to dictate the best practice.

After reading the article “COVID-19 in the Perioperative Period of Cardiovascular Surgery: the Brazilian Experience”, written by Gomes et al.^[1]^, we were inspired to collect and analyze our data, and share our institutional results. The mentioned article gathered data from 11 Brazilian centers and investigated mortality as a primary outcome and postoperative complications, ICU length of stay, and postoperative days of hospitalization as secondary outcomes.

We aimed to contribute by collecting data and reporting our service’s incidence and clinical and laboratory outcomes. Of 157 patients who underwent cardiovascular surgery from March 2020 to August 2021, six cases had a confirmed COVID-19 diagnosis by real-time reverse transcription-polymerase chain reaction (RT-PCR) in the perioperative period. Five urgent and one elective admissions were observed. Regarding clinical data, all patients were hypertensive, four had reduced left ventricular ejection fraction, and three had type 2 diabetes. Our patients’ mean European System for Cardiac Operative Risk Evaluation (EuroSCORE) II score was 5.52, varying from 0.55 to 13.64. The average time for COVID-19 diagnosis after surgery was 15.5 days, ranging from six to 29 days. Five patients underwent coronary artery bypass grafting and one underwent redo mitral and aortic valve replacement. The mean postoperative ICU stay was 5.67 days. After testing positive, five patients were readmitted to the ICU, and their mean length of stay was 11.6 days. Two patients required orotracheal intubation due to respiratory complications related to COVID-19, both progressing to death. Hospital stay had an average time of 30.33 days.

Regarding the laboratory evaluation, in most of them, a rise was seen in the C-reactive protein after surgery, and a much higher elevation was observed close to the COVID-19 diagnosis. The leucocyte count tended to increase after surgery and after the diagnosis was observed, a lower elevation was seen. The lymphocytes had a rapid peak after the COVID-19 diagnosis. The platelets did not exhibit a significant change.

The incidence of severe acute respiratory syndrome coronavirus 2 (SARS-CoV-2) in the perioperative period of cardiovascular surgery in our service was 3.82%, and the global mortality among those infected was 33.33%. Comparing to the study of Gomes et al.^[1]^, which found a mortality rate of 45.9% in the patients with a positive RT-PCR test within 10 days before or after surgery and of 27.3% in those with a positive diagnosis 10 days after surgery, our data are equivalent when divided into groups similarly to this study. There was a mortality rate of 33.3% in the first group and 33.3% in the second group. Another Brazilian study also found a mortality rate of 35.9%^[2]^. In conclusion, in our institution, patients who required reintubation had worse outcomes and a prolonged hospital length of stay.

During the pandemic, we had to deal with different scenarios and develop some alternative protocols; resilience was our most important tool to deliver the best care to our patients. There is a need for optimizing and adjusting the perioperative period of cardiovascular surgery for the COVID-19 era, such as the flow of care published by Mejia et al.^[3]^. Therefore, we thank the authors of the “COVID-19 in the Perioperative Period of Cardiovascular Surgery: the Brazilian Experience” article for their significant contribution to the medical community, and we also reaffirm the high risk, morbidity, and mortality of COVID-19 occurring in this period. During these hard days, analyzing and sharing knowledge save lives.
